# 

**Published:** 1882-09

**Authors:** 


### ARTICLE VI.

### AMERICAN PUBLIC HEALTH ASSOCIATION.

We have received the following notice from the Society of this important organization, and hope it will receive due attention from our readers:

BOSTON, AUGUST 10, 1882.

The American Public Health Association will hold its Tenth Annual Session at Indianapolis, Ind., commencing Tuesday, October 20, 1882.

Papers will be presented on the different action of disease in White and Black Races, The Removal of Excreta, Heredity, Sanitary Associations, Vaccination, Intermittent Fever in New England, Sanitary Organization, Cattle Disease, etc., etc., with Reports of Committees on the Prevention of Venereal Disease, Compulsory Vaccination, The Management of Epidemics, Statistics, Cattle Diseases, National Museum of Hygiene, Incorporation of the Association, and Necrology.

Terse, strong, practical papers on sanitary subjects are invited, and more time will be devoted to discussion than has been allotted at former meetings.

Papers offered to the Association become its exclusive property, and are copyrighted.

Gentlemen who propose to present papers, are respectfully requested to notify the Secretary at once of their intentions, and to give the titles of their papers that they may be assigned a place in the programme.

Present members of the Association are especially urged to every effort to increase its membership. Names proposed for membership should be sent to the Secretary.

Two proposed Amendments to the Constitution come up for action at this session.

It is expected that Vol. III. of the transactions will be ready for distribution at or before the meeting.

Information in regard to transportation will be furnished by the Indianapolis Local Committee at an early date, and by a later bulletin from the Secretary, if required.

By order of the Executive Committee,

AZEL AMES, JR.,

*Secretary.*

---

### ARTICLE VII.

### THE TRI-STATE MEDICAL SOCIETY.

As was stated in a previous number of this journal, the above named society holds its eighth annual session, at Terre Haute, Ind., on the 26th, 27th, and 28th of September, 1882. The following statement sent us by the Secretary may be of interest to such as desire to attend the meeting:

PLEASE NOTICE.

1. The sessions will be held in DOWLING HALL.

2. Hotel rates for members at the Terre Haute House, $2.00, reduced from $3.00; at the National Hotel, $2.00, reduced from $2.50. Any other accommodations necessary will be arranged for by the Local Reception Committee.

3. Arrangements for special rates have been made with the railroads centering here and their connections, as follows; Vandalia Line, I. & St. L., C. & E.I., E. & T. H., L. & N., Ills. Central, W., St. L. & P., one and one-third fare for round trip. The P., C. & St. L., Cincinnati Southern and I. B. & W. give round trip excursion rates by application to local agents.

☞ Members of the profession, desiring to take advantage of these arrangements will please apply to Dr. J. E. Link, Chairman of the Committee of Arrangements, who will furnish full particulars.

4. Members are requested to be present at the first session, as the regular business will begin without delay.

5. Each session will be called promptly.

6. Papers are limited by rule to twenty-five minutes.

7. Time for discussion will be given after each paper, or series of papers.

8. Authors, unavoidably absent, will send their papers to the Secretary during the first day of the meeting.

9. All physicians will, on their arrival, apply for tickets of membership issued by the Secretary.

10. Volunteer papers are solicited, and arrangements will be made for their presentation.

LOCAL COMMITTEE OF RECEPTION.—S. J. Young, J. D. Mitchell, L. J. Willien, W. H. Roberts, G. W. Crapo.

---

### PELLETURINUM TANNICUM.

The alkaloid pelleturinum, lately extracted from the *cortex radicis granate*, is, according to Dr. H. Witt (St. Petersburg *Medical Weekly Journal*, April, 1882), the most certain of all our remedies for tapeworm. In five cases in which, for a number of years, all the usual remedies had proved themselves to be unsuccessful, about twenty-two grains of pelleturinum tannicum, followed by a tablespoonful of castor-oil, had the effect that the dead tapeworm appeared unbroken in the stool. The remedy is easily administered, as it is perfectly tasteless.—*Med. and Surg. Reporter, Phila.*

### BOOKS AND PAMPHLETS RECEIVED.

### BOOKS.

The Medical Digest, or Busy Practitioner's Vade Mecum. Neal. Leger, Smith & Co., London.

Insanity and Imbecility. A Rational Materialistic Definition of, with the Medical Jurisprudence of Legal Criminality. Howard. Montreal: Dawson Bros.

Diseases of the Rectum and Anus. Kelsey. Wm. Wood & Co., Med. Lib. 1882. W. T. Keener.

Homœopathy; What is It? Palmer. Davis, Detroit.

Percussion Outlines. Cutler and Garland. Boston: Houghton, Mifflin & Co.

Manual of Diseases of the Skin. Bulkley. New York: Putnam's Sons. W. T. Keener.

Materia Medica and Therapeutics. Vol. II. Inorganic Substances. Phillips and Johnson. New York: Wm. Wood & Co., Med. Lib., May, 1882. W. T. Keener.

Transactions American Gynæcological Society, 1881. Vol. VI. Philadelphia: Lea's Son & Co. W. T. Keener.

Tenth Michigan Registration Report, Lansing, Mich.

Practical Medical Anatomy. Ranney. Wm. Wood & Co.

The Experimental Method in Medical Science. Dalton. New York: Putnam's Sons.

Labor Among Primitive Peoples. Engleman. St. Louis: Chambers & Co. W. T. Keener.

Mental Pathology and Therapeutics. Grusinger. Translated by Robertson and Rutherford, Edinburgh. New York: Wm. Wood & Co. W. T. Keener.

Contributions to Practical Gynæcology. Donaldson. W. T. Keener.

Clinical Lectures on Diseases of the Urinary Organs. Thompson. W. T. Keener.

### PAMPHLETS.

Uterine Anteversions and Anteflexions. Wing.

Trance, Muscle Reading and Allied Nervous Phenomena. Beard.

A Contribution to the Subject of Nerve-Stretching. Morton.

Antepartum Hour-glass Contraction of the Uterus. Smith.

Health in the Common Schools. Second Annual Report New York Board of Health.

Treatment of Uterine Fibroids by Iodine. Radcliffe.

Expert Testimony in the Whitaker Case. Piper.

An Ephemeris of Materia Medica, Pharmacy, etc. Squibb.

The Practice of Gynæcology in Ancient Times. Jenks. W. T. Keener.

A Further Contribution to the Treatment of Pulmonary Cavities. Pepper.

Tonsillotomy and its Complication by Hæmorrhage. Powell.

Two Cases of Hemiachromatopsia. Hayes.

The Condition of the Eyes in Two Cases of Fatal Anæmia. Bettman.

The Columbus Medical College Embroglio. Baldwin.

The Presence of the Micrococcus in the Blood of Malignant Measles. Keating. Trow & Co., N. Y.

Proceedings of the Connecticut State Medical Society, 1882. Blakiston, Son & Co.

---

Larrabee says: "Quackery consists in this, that while with the regular scientific physician all things are held in common, all truths are shared, quacks, by conspicuous words and advertisements, lead the people to believe that they possess ideas not known to the regular profession, and this alone is their hold upon the people whereby they gain a livelihood. When by chance the community looks in on the regular profession, they are surprised to find that the quack has nothing new, nothing that is not borrowed from legitimate medicine." In short, if all would be content to be known simply as doctor, there would be an end to schism. On no other ground can the honest regular practitioner meet in council the disciples of the healing art. This platform is as broad as accumulated knowledge and experience can make it. They alone are responsible for results who refuse to adopt and live by it.—*Detroit Lancet.*

### CLINICS.

MONDAY.

Eye and Ear Infirmary—1.15 p. m., Otological, by Prof. Jones; 2.15 p. m., Ophthalmological, by Prof. Hotz.

Mercy Hospital—2 p. m., Medical, Profs. Hollister and Quine.

Rush Medical College—3 p. m., Dermatological and Venereal, by Prof. Hyde.

Woman's Medical College—2 p. m., Dermatological and Venereal, by Prof. Maynard.

TUESDAY.

Cook Co. Hospital—2 to 4 p. m., Medical and Surgical Clinics.

Mercy Hospital—2 p. m., Surgical Clinic, by Prof. Andrews.

Woman's Medical College—10 a. m., Prof. Ingals.

WEDNESDAY.

Chicago Medical College—2 p. m., Eye and Ear, by Prof. Jones.

Rush Medical College—2 p. m., Medical, by Prof. Bridge; 3 p. m., Ophthalmological and Otological, by Prof. Holmes; 3:30 to 4:30 p. m., Diseases of the Chest, by Dr. E. Fletcher Ingals.

Woman's Medical College—2 p. m., Eye and Ear, by Dr. W. T. Montgomery; 3 p. m., Diseases of Children, by Prof. Chas. W. Earle.

Eye and Ear Infirmary—2.30 p. m., Dr. E. J. Gardiner.

THURSDAY.

Chicago Medical College—2 p.m., Gynæcological, Prof. Dudley.

Rush Medical College—2 p. m., Diseases of Children, by Prof. Knox; 3 p. m., Diseases of the Nervous System, by Prof. Lyman.

Eye and Ear Infirmary—2 p. m., Ophthalmological, by Dr. Hotz.

Woman's Medical College—3 p. m., Surgical, by Prof. Owens.

FRIDAY.

Cook County Hospital—2 to 4 p. m., Medical and Surgical Clinics, by Profs. J. H. Hollister and R. N. Isham.

Mercy Hospital—2 p. m., Medical, by Prof. Davis.

SATURDAY.

Rush Medical College—2 p. m., Surgical, by Prof. Gunn.

Mercy Hospital—2 p. m., Surgical Clinic, by Prof. Andrews.

Chicago Medical College—3 p. m., Neurological, Prof. Jewell.

Woman's Medical College—2 p. m., Gynæcological, by Prof. Stevenson.

Daily Clinics, from 2 to 4 p. m., at the Central Free Dispensary, and at the South Side Dispensary.

